# Use of leukocyte and platelet-rich fibrin (L-PRF) in periodontally accelerated osteogenic orthodontics (PAOO): Clinical effects on edema and pain

**DOI:** 10.4317/jced.52760

**Published:** 2016-04-01

**Authors:** Francisco Munoz, Constanza Jiménez, Daniela Espinoza, Alain Vervelle, Jacques Beugnet, Ziyad Haidar

**Affiliations:** 1 DDS, certified Oral and MaxilloFacial Surgeon, Professor, Faculty of Dentistry, Universidad de los Andes, Santiago, Chile; 2 DDS, certified Periodontist and Implantologist, BioMAT’X- Centro de Investigación Biomédica (CIB), Faculty of Dentistry, Universidad de los Andes, Santiago, Chile; 3 DDS, Oral and MaxilloFacial Surgeon, BioMAT’X- Centro de Investigación Biomédica (CIB), Faculty of Dentistry, Universidad de los Andes, Santiago, Chile; 4 DDS, certified Oral and Maxillofacial Surgeon, (BioMAT’X-CIB UAndes Consultant), Private practice, Marseille, France; 5 DDS, certified Orthodontist, (BioMAT’X-CIB UAndes Consultant), Private practice, Marseille, France; 6 DDS, certified Implantologist, Oral and Maxillofacial Surgeon (M.Sc.), M.B.A., PhD. Professor and Scientific Director, Faculty of Dentistry, UAndes. Founder and Head of BioMAT’X-CIB-PMI (Plan de Mejoramiento Institucional en Innovación), Universidad de los Andes, Santiago, Chile

## Abstract

**Background:**

Demand for shorter treatment time is common in orthodontic patients. Periodontally Accelerated Osteogenic Orthodontics (PAOO) is a somewhat new surgical procedure which allows faster tooth movement via combining orthodontic forces with corticotomy and grafting of alveolar bone plates. Leukocyte and Platelet-Rich Fibrin (L-PRF) possess hard- and soft-tissue healing properties. Further, evidence of pain-inhibitory and anti-inflammatory potential is growing. Therefore, this study explores the feasibility, intra- and post-operative effects of using L-PRF in PAOO in terms of post-operative pain, inflammation, infection and post-orthodontic stability.

**Material and Methods:**

A pilot prospective observational study involving a cohort of 11 patients was carried out. A Wilcko’s modified PAOO technique with L-PRF (incorporated into the graft and as covering membrane) was performed with informed consent. Post-surgical pain, inflammation and infection were recorded for 10 days post-operatively, while the overall orthodontic treatment and post-treatment stability were followed up to 2 years.

**Results:**

Accelerated wound healing with no signs of infection or adverse reactions was evident. Post-surgical pain was either “mild” (45.5%) or “moderate” (54.5%). Immediate post-surgical inflammation was either “mild” (89.9%) or “moderate” (9.1%). Resolution began on day 4 where most patients experienced either “mild” or no inflammation (72.7% and 9.1%, respectively). Complete resolution was achieved in all patients by day 8. The average orthodontic treatment time was 9.3 months. All cases were deemed stable for 2 years.

**Conclusions:**

L-PRF is simple and safe to use in PAOO. Combination with traditional bone grafts potentially accelerates wound healing and reduces post-surgical pain, inflammation, infection without interfering with tooth movement and/or post-orthodontic stability, over a 2 years period; thus alleviating the need for analgesics and anti-inflammatory medications.

** Key words:**Periodontally accelerated osteogenic orthodontics, leukocyte and platelet-rich fibrin, corticotomy, osteogenesis, grafts.

## Introduction

Demand for shorter treatment time with none to minimal side effects (i.e. root resorption, gingival recession, tooth decalcification, etc…) is a main request of adults seeking orthodontic treatment (1,2). Unlike children, adults have special biological conditions (i.e. slower cell mobilization and collagen conversion, increased risk of periodontal disease and almost inexistent alveolar and maxillary growth) which prevent speeding up treatment via conventional means (i.e. applying stronger forces) without increasing risk of hyalinization, among other complications (1). To overcome such limitations, different techniques are constantly explored and developed over the years, to accelerate tooth movement, with surgical endeavors reporting the highest success rates. Indeed, such surgical attempts date back to 1959, when Kole theorized that cortical bone plates were the main resistance for tooth movement. Thereby, a corticotomy/osteotomy procedure which selectively sectioned the plates, was presented with promising results. Despite, it was not widely accepted due to subapical horizontal cuts penetrating full thickness of the alveolar ridge (1). Subsequent technical modifications included conservative corticotomies alongside the discovery that periodontal health could be maintained if the vertical cuts avoided the crestal bone area (1). This led the Wilcko brothers (a periodontist and an orthodontist) to introduce, in 2001 a new technique for surgically-assisted tooth movement in orthodontics (3-6). Their technique combined classic corticotomies/osteotomies of the alveolar bone with the use of bone grafts in order to maintain and increase the thickness of the cortical plates into which teeth were moved. Wilcko’s novel “Periodontally Accelerated Osteogenic Orthodontic or PAOO” technique gained acceptance and popularity given its safe, predictable and effective results as well as benefits versus traditional orthodontics; which included: accelerated differential tooth movement, reduced treatment time, less root resorption, enhanced expansion, increased traction of impacted teeth, increased post-treatment stability and increased robustness of the periodontum (including recently reported increase in the width of keratinized gingiva) (1,4,7). With grafting, no more limits regarding pre-existing alveolar volume existed, allowing the teeth to be moved 2 to 3 times more (distance) and in almost 1/3rd of the conventional/traditional time (1,2,7). Today, main indications for PAOO include moderate to severe crowding, Class II malocclusions requiring expansion and/or extractions, mild Class III malocclusions, extrusion for open bite and intrusion for deep bite (1,7). Recently, PAOO has also been suggested to reduce the need and extension of orthognathic surgery in specific patients, opening new and exciting frontiers and possibilities within maxillofacial surgery (1,4,8). The rapid tooth movement and stability, as a result of PAOO, has been attributed to a localized and temporal osteoporosis-like/increased turnover state of the bone, referred to as Regional Acceleratory Phenomenon or RAP (1). Briefly, RAP is a natural event within the bone healing process which usually follows fracture, osteotomy and/or grafting. The PAOO procedure, therefore, involves the activation and recruitment of precursor cells into the wounded/injured site, leading to subsequent two to tenfold increase in hard and soft tissue healing (9). In PAOO, RAP begins within few days of the surgical intervention, peaks at 1 to 2 months post-surgery and usually lasts up to 4 months (though 6 and up to 24 months may be necessary to completely subside). However, as long as tooth movement continues, RAP follows (1). As any healing-related event, RAP requires a delicate combination of progenitor cells, extracellular signaling molecules and adequate extracellular matrix in order to succeed. In this “tissue engineering” context, biomaterials that support and enhance the regenerative process may further improve the clinical outcomes of PAOO while reducing side-effects (which often include: slight interdental bone loss, loss of attached gingiva, periodontal defects, subcutaneous hematomas and postoperative pain and swelling for several days) (1). Leukocyte and Platelet-Rich Fibrin or L-PRF is a second generation platelet hydrogel obtained through the simple and rapid centrifugation of whole blood samples in absence of anti-coagulants and bovine thrombin (10,11). The biomaterial is characterized by a dense fibrin mesh (similar to natural extracellular matrix) and rich platelet-, leukocyte-, growth factor- and stem cell-content (exceeding that of conventional blood clots and Platelet-Rich Plasma or PRP) (12). Accumulating evidence (13,14) demonstrates that L-PRF membranes actively produce and release abundant concentrations of growth factors and cytokines for up to 28 days post-preparation; exerting: (a) dose-dependent osteoinductive effects over osteoblasts, periodontal ligament cells and bone marrow mesenchymal stem cells (which may be further increased via combination with autologous bone) as well as potential (b) anti-inflammatory, (c) anti-infective and (d) pain inhibitory properties (15); all of which are attractive properties suitable for incorporation into PAOO. Here in, we aimed to explore the clinical feasibility or effect of preparing and using L-PRF in PAOO in terms of post-operative inflammation, pain, infection and short-term orthodontic stability, as the study outcomes.

## Material and Methods

-Study design and population

A cohort observational study was designed involving 11 patients who visited our clinics between June 2013 and June 2015. Inclusion criteria: (a) patients in need of orthodontic treatment whom desired a shorter treatment time and (b) patients whom (according to the Orthodontic and Periodontal specialists and consultants) were suitable candidates for PAOO. Exclusion criteria: (a) patients with systemic health illnesses incompatible with undergoing surgery and (b) patients with active periodontal disease. Suitability for PAOO was confirmed after an extensive series of examinations which included: mounted model casts, radiographic analysis (panoramic and lateral radiographs), cephalometric analysis (Vicker Sassouni) and both, intra and extra-oral photographs. This study complies with the guidelines of the World Medical Association-Helsinki Declaration 2000 for biomedical research and was reviewed, approved and supervised by the Ethics committee (Medicine/Dentistry Schools) of the University of the Andes in Santiago, Chile.

-L-PRF preparation.

Peripheral blood samples were drawn into 10 mL glass-coated tubes without anti-coagulants (6-8 tubes per patient). Samples were immediately table-top centrifuged at 3000 rpm for 10 mins, according to the protocol developed by Choukroun *et al.* in 2001 (11). Clots were then carefully separated from red blood cell precipitants using scissors. Half of the clots were minced and incorporated into the bone graft while the other half prepared as a membrane using L-PRF box.

-Surgical procedure

All surgeries (N=11) were performed by the same team. Following an informed and written consent, a modified “Wilcko” technique incorporating the use of L-PRF (60 to 80 mL of blood per patient) as grafting material and barrier membrane, with antibiotic treatment. After administration of anesthesia, full-thickness envelope flap is carefully raised preserving the interdental papillae. Vertical corticotomy patterns are performed using either rotary (ESCAROM n°2 round burs at 500 rpm) or piezoelectric (ES-CAROM, BS1 tip) instruments. Cuts extend from 2 mm below the bone crest to approximately 2-3 mm past the teeth apices, penetrating 1.5 to 2 mm into the cortical plate until reaching the cancellous bone [evidence suggest that corticotomy pattern, depth, extent and means of creation (either rotary or piezoelectric) are not crucial factors for the success of PAOO (4)]. After controlling the bleeding, minced pieces of L-PRF (4 clots) are grafted with a 3:2 combination of Puros cortico-cancellous® (Zimmer Dental Inc, CA, USA) and Bio-Oss® (Geistlich Pharma North America Inc, Princeton, USA), respectively; with the addition of two (250 mg each) Metronidazole capsules (total of 500 mg mixed into the graft). It is noteworthy that final graft volume varies according to individual patient needs in terms of tooth movement, bone thickness and labial support. L-PRF membranes (3 to 4 per patient) are then placed over the graft in order to contain it on the buccal aspect of the alveolar plate. Finally, the flap is repositioned and sutured using tension-free trans-papillary Donati Blair stitches (4-0 vicyrl). The recommended post-operative management regimen typically includes: Chlorhexidine mouthwash (0.12%, every 6 hrs for 10 days), Amoxicillin (2 g every 24 hrs for 7 days), Cortisol (30 mg every 24 hrs for 2 days) and Ibuprofen (400 mg every 8 hrs, as needed). Sutures are removed ~ 10 days post-operatively.

-Data collection

All 11 patients were monitored at days 1, 2, 4, 8 and 10 post-op. At each time-point, complete clinical evaluations with intra- and extra-oral photographs were performed. Post-surgical inflammation, infection and pain were recorded using clinical parameters and patient feedback. Briefly, inflammation was measured by means of presence and extension of clinical edema using a customized scale. Infection was measured as either “present” (+) or “absent” (-) upon presence of abscesses, suppuration and/or fistulae. Finally, quantification and measurement of pain was made by patients via a customized scale to report extent and need for post-operative analgesics.

-Orthodontic procedure

All patients (N=11) were treated by the same orthodontist. Full maxillary and mandibular braces with self-ligating brackets were placed 7 days pre-surgery, followed by a 0.12, 0.14 or 0.16 NiTi arch protocol, according to the individual need of each patient; with activation every two weeks. Total treatment time (from placement of appliances until removal) and post-treatment stability were documented using rigid night guard splints. Stability was recorded as either “stable” (if the splint fitted correctly) or “unstable” (if it did not fit). No post-orthodontic contentions were utilized.

-Statistical analysis

Descriptive statistics for baseline patient characteristics (age, gender, diagnosis, response to treatment and post-orthodontic stability) were applied. Further testing was deemed un-necessary.

## Results

**Figure 1:**
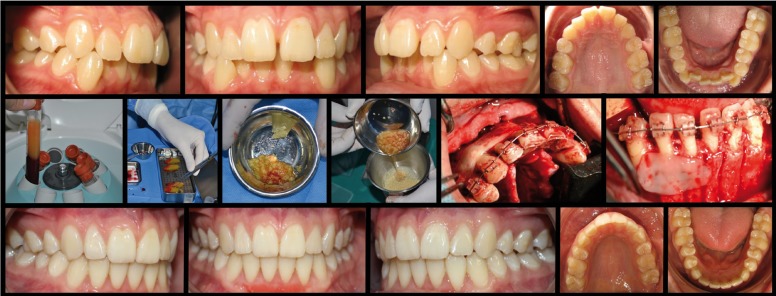
L-PRF preparation.

Eleven patients were enrolled in this study (3 males and 8 women; average age: 34.8 years). All subjects completed the 2-years follow-up. Baseline demographic data and diagnosis are summarized in  Table 1. L-PRF preparation was done intra-operatively (~15 minutes) while performing corticotomy patterns; (Fig. 1). From clinician’s stand-point, the biomaterial was simple to prepare, handle and suture. In this technique, minced pieces of L-PRF mixed into the graft provide increased stability when placed on the buccal aspect of the surgical sites. Further, the L-PRF covering membranes supplied additional stability as well as graft protection against exposure and contamination. After treatment with L-PRF, all patients experienced accelerated flap healing with no signs of infection or adverse reactions. Postoperative pain (based on post-surgical need of NSAIDs) was rated either as “mild” (45.5% of patients, analgesic consumption up to 48 hrs post-op) or “moderate” (54.5% of patients, analgesic consumption up to 6 days post-op). No “severe” pain was reported (need of analgesic consumption for more than 6 days) at any point of the study; ( Table 2). All patients showed clinical signs of edema on the first day. Inflammation (according to our customized scale on clinical intra- and extra-oral edema) was either “mild” (89.9%, edema limited to the buccal aspect of the surgical site) or “moderate” (9.1%, edema on buccal aspect of the surgical site which also extended to neighbor intra-oral structures). Inflammatory peak was reached by day 2 with inflammation either increasing to “moderate” levels (72.7% of patients) or maintaining its original extent (“moderate” in 9.1% of patients and “mild” in 18.2%). Edema resolution begun by day 4 with most patients (72.7%) exhibiting decreased inflammation levels returning to a “mild” level of inflammation. Only 1 patient (9.1%, Patient N° 11) experienced complete resolution (no clinical signs of intra- or extra-oral edema). Another subject (9.1%, Patient N° 8) presented no changes in his inflammatory levels from days 1 to 4, sustaining a “moderate” level inflammation. Overall, complete resolution of the inflammatory process was achieved by day 8 and continued on day 10. It is noteworthy that none of the patients presented “severe” inflammation (presence of intra- and extra-oral clinical signs of edema); ( Table 3). Finally, the average time for orthodontic treatment was 9.3 months (dispersion ranging from 3 to 18 months) and all cases maintained stability for at least 2 years post-surgery. It is worth mentioning that in this pro-cohort study, patients were generally satisfied and neither gender nor age seem to have any detrimental effect on the treatment time, result or post-treatment stability.

**Table 1:**
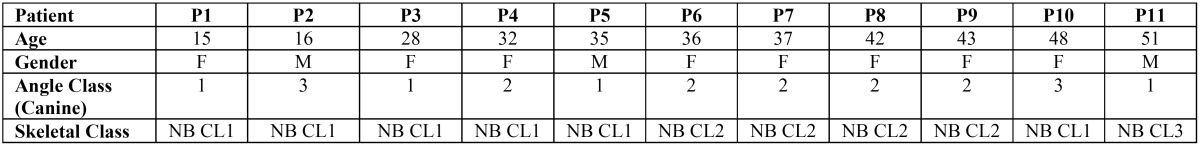
Demographic data and clinical diagnosis of patients (N=11) involved in the study.

**Table 2:**
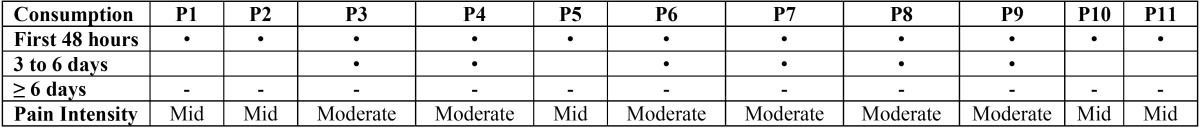
Post-operative self-reported need for analgesics and overall pain intensity.

**Table 3:**
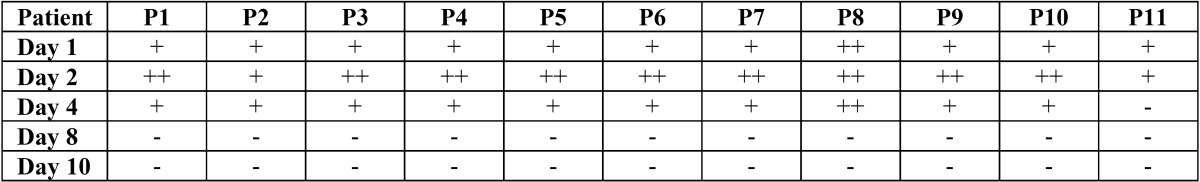
Post-operative edema (after treatment with L-PRF).

## Discussion

PAOO is a new and un-explored territory in Dentistry. Most of accumulating evidence generates from observational and ca-se-control studies. L-PRF is a 2nd generation platelet concentrate vastly used in oral and maxillofacial surgery due to its angiogenic, osteogenic and wound healing properties (15,16). To the best of our knowledge, a single report of L-PRF application within PAOO (4) exists in the literature. The article uses L-PRF to increase bone graft stability over the surgical site (also evidenced in this study) without any further description of the method of application or evaluation of the resulting intra- and post-operative clinical effects (4). Hence, the present cohort prospective study not only explored these questions but also proposes a methodological technique for L-PRF preparation and application suitable for PAOO (a modified version of Wilcko’s PAOO) which includes the use of 4 L-PRF clots as grafting material (minced and mixed into a 3:2 Puros®/Bio-Oss® graft) and 3 to 4 L-PRF membranes for coverage. An additional intra-operative medication of the graft with metronidazole to protect the graft from the un-avoidable bacterial contamination during surgery, was done. Such methodological and technical decisions were deduced from another study (17) which evaluated the use of L-PRF in complex maxillary reconstructions (resembling PAOO in duration and wound extensity). Here in, the wound healing and osteogenic properties of L-PRF were previously evaluated clinically by means of observing healing of the flap and post-orthodontic stability (which has been associated to post-treatment bone thickness (4) and thus proposed as an indirect non-invasive parameter for assessing the osteogenic potential of L-PRF). Our results regarding accelerated flap healing and long-term post-treatment stability (up to 2 years) not only support the wound healing and osteogenic properties of L-PRF, yet also suggest that the healing capacity of L-PRF may exceed the limits reported. Indeed, the surgical wound in PAOO greatly exceeds both in extension and complexity that of implant, periodontal and extraction-socket defects (the main sources of human clinical trial evidence for L-PRF literature in Dentistry). The enhanced soft and hard tissue healing observed in this study may be attributed to the intrinsic properties of the L-PRF fibrin matrix, cellular- and growth factor-rich content, as well as the use of L-PRF as a covering membrane. It is a well-established basic surgical principle that for healing, adequate blood supply and protection of the surgical site are necessary. In addition, contemporary tissue engineering concepts propose the need of an adequate extracellular matrix, presence of progenitor cells and appropriate levels of signaling molecules. Evidently, the corticotomies induced within PAOO assure a rich and vascularized bed for the graft whereas L-PRF as cover membrane has been clinically associated with increased gingival remodeling and thickness (15,17). This combinatorial approach seems to reduce the risk of tear/dehiscence of the flap with subsequent graft exposure and contamination, providing an optimal environment for wound healing. On the other hand, the sponge-like architecture of the biomaterial provides an ideal scaffold for free cell migration into the surgical site; while the release of growth-factors for up to 28 days post-surgery, provides the continuous long-term stimuli required for chemotaxis and osteogenic differentiation of osteoblasts, periodontal ligament cells and bone-marrow mesenchymal stem cells (15). Furthermore, the presence of CD34+ stem cells within the L-PRF (18) provides availability of progenitor cells to augment the healing process. On the other hand, recently accumulated evidence including human trials using L-PRF in periodontal surgery associated L-PRF use with decreased post-surgical pain and inflammation (19,20). This concept is relatively new within the L-PRF literature and thus is still not fully understood. Plausible biological mechanisms behind this phenomenon (as well as for reduced post-surgical risk of infection) have been attributed to the leukocyte-rich content which produces: 1) anti-nociceptive molecules (such as b-endorphin, matenkephalin and dynorphin-a) and 2) anti-inflammatory cytokines (such as IL-4, IL-10 and IL-13) (20). In this study, post-surgical pain (measured by means of need for consuming non-steroidal anti-inflammatory drugs or NSAIDs) and clinical inflammation (evaluated in terms of presence and extension of intra and extra-oral edema) was reduced with the use of L-PRF. Finally, despite the advantages of PAOO when compared to traditional orthodontics (1,4), patients are often discouraged due to fear of undergoing surgery with additional costs and post-operative discomfort (mainly pain, inflammation and risk of infection). In this context, L-PRF may be a cost-effective way to increase patient acceptance and other major surgical interventions (i.e. distraction osteogenesis and orthognathic surgery, for example). An interesting topic for future investigation is to determine whether L-PRF reduces the need for post-surgical NSAIDs. Avoiding such drugs not only could further improve tooth movement and post-orthodontic stability in PAOO (as NSAIDs have been associated with interference of bone metabolism and turnover), but also reduce the drug-related adverse effects in patients, thereby growing patient acceptance of maxillofacial orthognathic surgical procedures.

## Conclusions

Findings of this study suggest that L-PRF is a simple, malleable and safe biomaterial suitable for use in PAOO. Combination with conventional particulate bone grafts seems to reduce post-operative inflammation, pain and risk of infection without interfering with tooth movement or post-orthodontic stability (<2 years). This is critical since commonly-used analgesic and non-steroidal anti-inflammatory drugs are known to interfere with bone healing and tooth movement. Other drugs including aspirin, diclofenac, ibuprofen, indomethacin and celecoxib, reduce tooth movement via decreasing bone turnover due to orthodontic forces. Acetaminophen is the only analgesic drug with no documented negative effects bone physiology and metabolism. L-PRF might be a useful and effective alternative. Randomized multi-center prospective trials with more patients and longer follow-ups are currently undergoing. Cost-effectiveness and psycho-socio-economics are important aspects involved in the study design. To the best of knowledge, this is first report investigating the feasibility, clinical impact and short-term post-orthodontic stability of using L-PRF in PAOO. It introduces a modified PAOO technique. Our group is currently investigating the potential of incorporating oral-derived mesenchymal stem cells or growth-factor embedded nanoparticles within the L-PRF, as bio-scaffolds, to further boost, with predictability, bone formation, tooth movement ability, treatment time and post-orthodontic stability, in PAOO. Our research extends to investigate the potential of L-PRF in reducing the need for prescription drugs following invasive surgical procedures such as third molar extraction and cysts resections.
